# Cilostazol and Functional Outcomes in Poor-Grade Aneurysmal Subarachnoid Hemorrhage: A Multicenter Retrospective Analysis

**DOI:** 10.3390/jcm15166386

**Published:** 2026-08-18

**Authors:** Jin Kikuchi, Yusuke Otsu, Sosho Kajiwara, Kimihiko Orito, Masaru Hirohata, Kiyohiko Sakata, Katsumi Takizawa, Yoji Tanaka, Masaki Chin, Hiroki Kurita, Akihiro Hirayama, Koreaki Irie, Kenichiro Suyama, Ichiro Nakahara, Nobutaka Horie, Motohiro Morioka, Fusao Ikawa

**Affiliations:** 1 Department of Neurosurgery, Kurume University School of Medicine, Kurume 830-0011, Fukuoka, Japan; ohtsu_yuusuke@med.kurume-u.ac.jp (Y.O.); kajiwara_soushou@med.kurume-u.ac.jp (S.K.); kiyo@med.kurume-u.ac.jp (K.S.);; 2 Department of Neurosurgery, Japanese Red Cross Asahikawa Hospital, Asahikawa 070-0061, Hokkaido, Japan; 3 Department of Neurosurgery, Kyorin University School of Medicine, Mitaka 181-8611, Tokyo, Japan; yoji-tanaka@ks.kyorin-u.ac.jp; 4 Department of Neurosurgery, Kurashiki Central Hospital, Kurashiki 710-0052, Okayama, Japan; 5 Department of Cerebrovascular Surgery, Saitama Medical University International Medical Center, Hidaka 350-1298, Saitama, Japan; 6 Department of Neurosurgery, Tokai University School of Medicine, Isehara 259-1193, Kanagawa, Japan; 7 Department of Neurosurgery, Japan Red Cross Medical Center, Shibuya-ku 150-8935, Tokyo, Japan; 8 Department of Neurosurgery, Fujita Health University, Toyoake 470-1192, Aichi, Japan; qqpp8dx9n@icloud.com; 9 Department of Neurosurgery, Fukuoka Shin Mizumaki Hospital, Onga 807-0051, Fukuoka, Japan; 10 Department of Neurosurgery, Graduate School of Biomedical and Health Sciences, Hiroshima University, Hiroshima 734-0037, Hiroshima, Japan; 11 Department of Neurosurgery, Shimane Prefectural Central Hospital, Izumo 693-8555, Shimane, Japan; fikawa-nsu@umin.ac.jp

**Keywords:** cilostazol, delayed cerebral ischemia (DCI), prognostic factors, subarachnoid hemorrhage (SAH)

## Abstract

**Background/Objectives**: Poor-grade aneurysmal subarachnoid hemorrhage (SAH; WFNS grades 4–5) carries a historically dismal prognosis, driven by early brain injury and delayed cerebral ischemia (DCI). Despite advances in neurocritical care, effective pharmacological strategies for this vulnerable population remain limited. This multicenter study investigated prognostic factors for functional recovery, with particular emphasis on the potential benefit of adjunctive cilostazol, a phosphodiesterase-3 inhibitor with vasodilatory and antiplatelet properties. **Methods**: This multicenter retrospective cohort study analyzed 394 patients with WFNS grade 4–5 aneurysmal SAH treated at 10 institutions between 2018 and 2024. The primary outcome was favorable functional status (modified Rankin Scale 0–2) at 6 months. Multivariable logistic regression was performed to identify independent predictors of outcome. Sensitivity analyses were also performed to assess the potential influence of missing 6-month outcome data. **Results**: Six-month outcomes were available for 318 patients (80.7%), of whom 107 (33.6%) achieved favorable recovery. Patients with favorable outcomes were significantly more likely to have received cilostazol than those with unfavorable outcomes (70.1% vs. 55.5%; *p* = 0.016). In multivariable logistic regression, cilostazol use remained associated with a favorable outcome (odds ratio [OR] 2.29; 95% confidence interval [CI] 1.01–5.22; *p* = 0.048) after adjustment for age (OR 0.95; *p* < 0.001), WFNS grade 5 (OR 0.46; *p* = 0.012), ventricular drainage, and cerebral infarction. Sensitivity analyses, including propensity score–weighted and missing-outcome analyses, yielded directionally favorable estimates but were not statistically significant. **Conclusions**: Despite the severity of the initial insult, one-third of patients with available 6-month follow-up achieved functional independence. Cilostazol use was associated with favorable functional outcome in the primary analysis, although statistical significance was not retained in sensitivity analyses addressing missing outcomes. These findings suggest a potential therapeutic signal of cilostazol in poor-grade aneurysmal SAH, warranting prospective validation.

## 1. Introduction

Aneurysmal subarachnoid hemorrhage (SAH) remains one of the most catastrophic forms of cerebrovascular disease, with substantial mortality and long-term disability despite modern advances in neurosurgical and neurocritical care [1]. Patients presenting with poor neurological grade—specifically World Federation of Neurosurgical Societies (WFNS) grades 4 and 5—constitute a distinct and particularly vulnerable subgroup [2]. While survival rates have improved, functional independence remains elusive, reflecting the profound impact of early brain injury (EBI) and secondary insults [3,4].

Delayed cerebral ischemia (DCI), typically occurring between 4 and 14 days after aneurysm rupture, represents one of the few potentially modifiable determinants of neurological deterioration [5]. Preventive strategies have therefore focused largely on vasospasm mitigation [5]. Standard therapy includes nimodipine and maintenance of euvolemia, while in Japan, fasudil hydrochloride and the endothelin receptor antagonist clazosentan are widely used [1,6]. While these agents effectively reduce angiographic vasospasm, this success has not consistently translated into improved functional outcomes, particularly in patients with poor-grade SAH [2,7].

This apparent dissociation underscores the multifactorial nature of injury in severe SAH [2]. Beyond large-vessel vasospasm, microcirculatory dysfunction, platelet activation, endothelial injury, and inflammatory cascades may critically influence neurological recovery [4,8]. In this context, cilostazol—a phosphodiesterase-3 inhibitor with vasodilatory, antiplatelet, and putative endothelial-protective properties—has emerged as a candidate adjunctive therapy [9]. Randomized studies and meta-analyses have suggested reductions in symptomatic vasospasm and cerebral infarction with cilostazol use [10,11]. However, these investigations have predominantly enrolled patients with good neurological grade, and the evidence base for its use in poor-grade SAH remains limited [10,11,12].

Critically, the subgroup at highest risk—patients with poor-grade SAH—remains underrepresented in high-quality clinical trials [10,11,12]. There is a paucity of data regarding the real-world efficacy of pharmacological DCI prophylaxis in this vulnerable population, specifically whether the pleiotropic effects of cilostazol translate into functional benefits despite the severity of the initial insult [13].

In this context, we conducted a multicenter retrospective cohort study to (1) identify prognostic factors associated with functional recovery in poor-grade SAH and (2) evaluate the association between cilostazol use and 6-month functional outcome in routine clinical practice.

## 2. Materials and Methods

### 2.1. Ethical Considerations

This multicenter study was approved by the ethics committees or Institutional Review Boards (IRBs) of all participating institutions. The representative study protocol was initially approved by the Shimane Prefectural Central Hospital Ethics Committee in 2022 (Approval No. R22-020) and subsequently underwent continuing review, with the most recent approval granted on 5 June 2025. The requirement for written informed consent was waived owing to the retrospective design, using an institutional opt-out approach. The study was conducted in accordance with the Declaration of Helsinki and is reported in accordance with the Strengthening the Reporting of Observational Studies in Epidemiology (STROBE) guidelines [14,15].

### 2.2. Study Design and Patient Population

This multicenter, retrospective cohort study involved 10 neurosurgical institutions in Japan. Medical records of patients admitted with aneurysmal SAH between 2018 and 2024 were reviewed. Inclusion criteria were as follows: (1) diagnosis of aneurysmal SAH confirmed by computed tomography (CT) or lumbar puncture; (2) poor neurological grade on admission, defined as WFNS grade 4 or 5; and (3) treatment of the ruptured aneurysm by surgical clipping or endovascular coiling. Patients who died before aneurysm treatment and those with non-aneurysmal SAH were excluded. A total of 394 patients met the inclusion criteria. The choice of aneurysm treatment (clipping or coiling) was made at the discretion of the attending neurosurgical team based on aneurysm characteristics and the patient’s clinical condition.

### 2.3. Treatment Protocols

All patients received standard neurocritical care management according to contemporary guidelines. Prophylactic strategies for DCI varied among institutions but generally consisted of adequate hydration and pharmacological therapy. Pharmacological agents included fasudil hydrochloride (30 mg intravenously three times daily), clazosentan (10 mg/h continuous intravenous infusion), cilostazol (100 mg enterally twice daily), statins, and intrathecal milrinone, depending on institutional protocols and physician discretion. Cilostazol was used as a prophylactic therapy for delayed cerebral ischemia and was generally administered as part of the management strategy during the vasospasm-prone period. Treatment was generally initiated after aneurysm securing. When oral intake was not feasible because of impaired consciousness, cilostazol was administered enterally through a nasogastric tube. Treatment was typically continued during the vasospasm-prone period unless contraindications or adverse events necessitated discontinuation. However, the exact initiation date, treatment duration, cumulative dose, and discontinuation timing were not systematically recorded for individual patients. Cerebrospinal fluid (CSF) drainage (ventricular, cisternal, or lumbar) was performed as clinically indicated for hydrocephalus or subarachnoid clot clearance at the discretion of the treating physician.

### 2.4. Outcomes and Definitions

The primary outcome was functional status at 6 months after SAH onset, assessed using the modified Rankin Scale (mRS). Favorable outcome was defined as mRS 0–2.

Secondary outcomes included angiographic vasospasm, symptomatic vasospasm, and cerebral infarction.

Angiographic vasospasm was defined as ≥50% arterial narrowing compared with baseline imaging [16]. Symptomatic vasospasm was defined as new focal neurological deficits and/or a ≥2-point decrease in the Glasgow Coma Scale lasting ≥1 h in the presence of angiographic vasospasm, after exclusion of alternative causes [17].

Cerebral infarction was defined as a new infarct detected within 6 weeks after SAH onset that was absent on early postoperative imaging and was not considered attributable to a surgical or endovascular procedural complication based on the available imaging and clinical records [17]. Because the presumed mechanism of each remaining infarction was not systematically recorded, reliable differentiation between vasospasm-related infarctions and other nonprocedural infarctions was not possible. Cilostazol exposure was defined as documented administration of cilostazol as part of DCI prophylaxis during the index hospitalization. Because the registry did not systematically capture the exact initiation date, treatment duration, cumulative dose, or discontinuation timing, cilostazol exposure was analyzed as a binary variable. Accordingly, timing-, duration-, dose-, and exposure–response relationships could not be assessed.

### 2.5. Statistical Analysis

Continuous variables are presented as mean ± standard deviation (SD), and categorical variables as numbers and percentages. Comparisons between groups were performed using Student’s *t* test for continuous variables and the chi-square test or Fisher’s exact test for categorical variables. Functional outcome analyses were conducted in patients with available 6-month mRS data. Variables associated with functional outcome at a two-sided *p* value of <0.05 in the univariable analyses were included in the multivariable logistic regression model. Multicollinearity among the covariates included in the multivariable logistic regression model was assessed using variance inflation factors (VIFs). A VIF greater than 5 was considered indicative of potentially important multicollinearity. Baseline and treatment characteristics were compared between patients with and without available 6-month functional outcome data. To assess the potential influence of missing outcomes, multiple imputation was performed using available demographic, disease-severity, and treatment-related variables. Multivariable logistic regression was repeated in each imputed dataset, and the estimates were combined using Rubin’s rules. As an additional conservative boundary analysis, all patients with missing 6-month outcomes were classified as having an unfavorable outcome, and the multivariable model was repeated using the same covariates as in the primary analysis. To account for potential clustering of patients within participating institutions, an additional mixed-effects logistic regression model was fitted with study center included as a random intercept. The fixed-effect covariates were identical to those included in the primary multivariable logistic regression model. This analysis was performed as a sensitivity analysis of the primary conventional logistic regression model. To address potential confounding by indication, propensity score–weighted analyses were performed among 271 patients with available 6-month functional outcome data and complete information for the variables included in the propensity score model. A propensity score for cilostazol use was estimated using logistic regression based on baseline variables available before or at the time of treatment selection, including age, sex, WFNS grade 5, Fisher grade 4, hypertension, diabetes mellitus, and aneurysm treatment modality. Cerebral infarction was not included because it occurred after treatment selection and may represent an intermediate variable. Propensity score weighting targeting the average treatment effect among the treated (ATT) was performed, together with an additional analysis targeting the average treatment effect in the overall study population (ATE). Covariate balance before and after weighting was assessed using absolute standardized mean differences, with values below 0.10 considered indicative of acceptable balance. The association between cilostazol use and favorable 6-month functional outcome was evaluated using weighted logistic regression with robust standard errors. To assess the potential influence of concomitant DCI-preventive medications, the frequencies of the combinations of fasudil, cilostazol, clazosentan, and intrathecal milrinone and their corresponding favorable 6-month functional outcome rates were examined descriptively among the 271 patients included in the complete-case multivariable analysis. Because cilostazol monotherapy and several individual treatment combinations included very small numbers of patients, formal comparisons and separate multivariable models for each drug combination were not performed. The complete distribution of treatment combinations is provided in Supplementary Table S2. Instead, an additional multivariable logistic regression model was constructed by adding concomitant fasudil, clazosentan, and intrathecal milrinone use to the covariates included in the primary model. Landmark and time-dependent exposure analyses were not feasible because exact patient-level dates of cilostazol initiation and discontinuation were unavailable. All statistical analyses were performed using JMP^®^ 19 (JMP Statistical Discovery LLC, Cary, NC, USA).

## 3. Results

### 3.1. Patient Characteristics

Baseline characteristics of the 394 patients with poor-grade aneurysmal SAH are summarized in Table 1. The mean age was 67.2 ± 13.6 years, and 69.0% were female. On admission, 136 patients (34.5%) were classified as WFNS grade 4 and 258 (65.5%) as grade 5. Fisher grade 4 hemorrhage was present in 147 patients (37.3%). Hypertension was observed in 41.4% and diabetes mellitus in 8.9% of the cohort. Surgical clipping was performed in 210 patients (53.3%), whereas 184 (46.7%) underwent endovascular coiling. Regarding acute-phase DCI prophylaxis, cilostazol was administered in 250 patients (63.5%), fasudil in 231 (58.6%), and clazosentan in 142 (36.0%). Ventricular drainage was required in 162 patients (41.1%), and lumbar drainage in 134 (34.0%).

### 3.2. Clinical Outcomes

Among the 394 patients, angiographic vasospasm occurred in 20.1%, and symptomatic vasospasm in 11.2%. Cerebral infarction was identified in 86 patients (21.8%). Six-month functional outcome data were available for 318 patients (80.7%). Among these, 107 patients (33.6%) achieved a favorable outcome (mRS 0–2).

**Figure 1:**
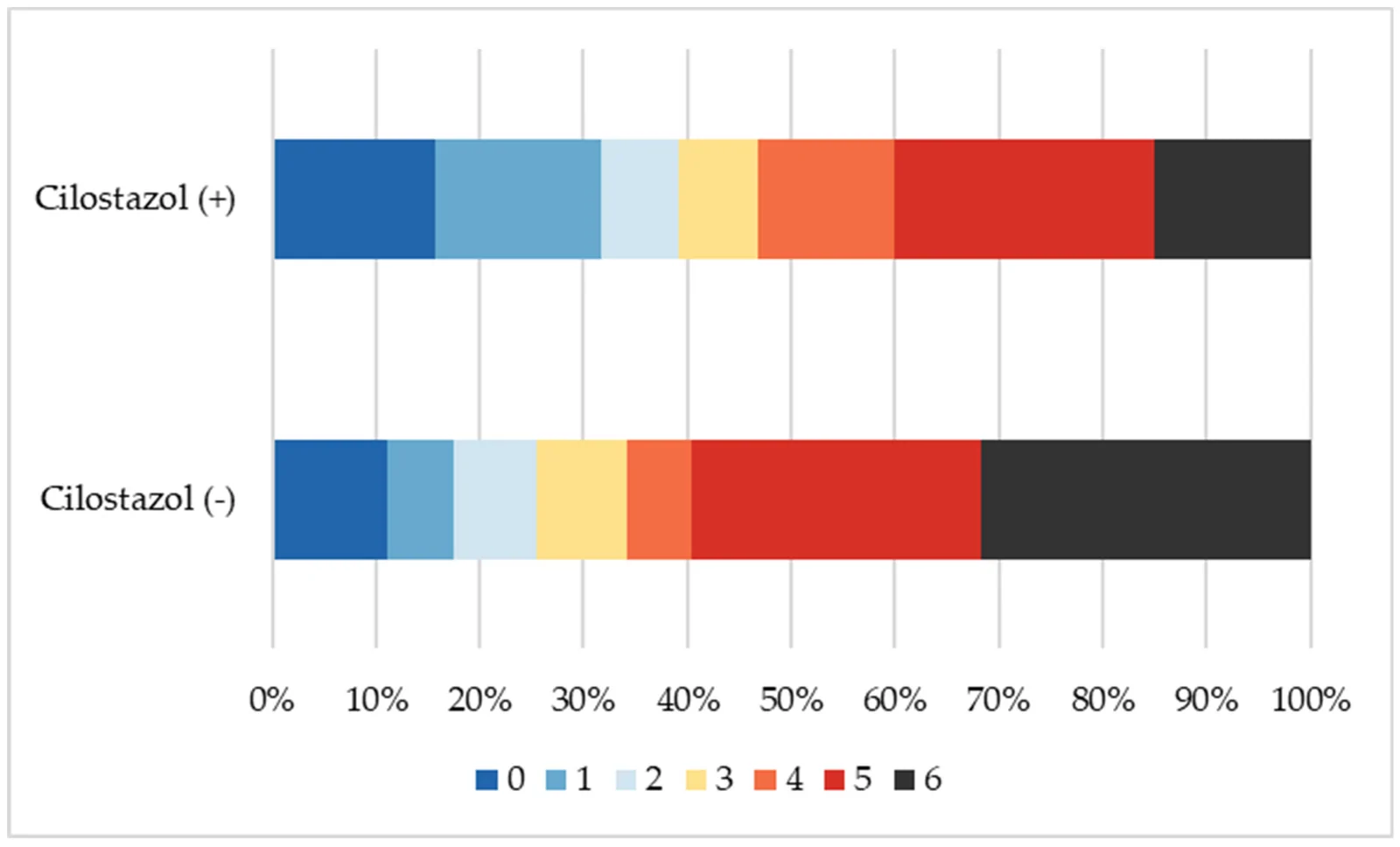
Distribution of 6-month modified Rankin Scale (mRS) scores according to cilostazol exposure. The distribution of mRS scores is shown for patients who received cilostazol and those who did not.

To further explore differences in functional recovery, outcomes were stratified according to cilostazol exposure. As shown in Figure 1, the distribution of 6-month mRS scores differed descriptively between patients who received cilostazol and those who did not.

### 3.3. Functional Outcomes and Sensitivity Analyses

**Figure 2:**
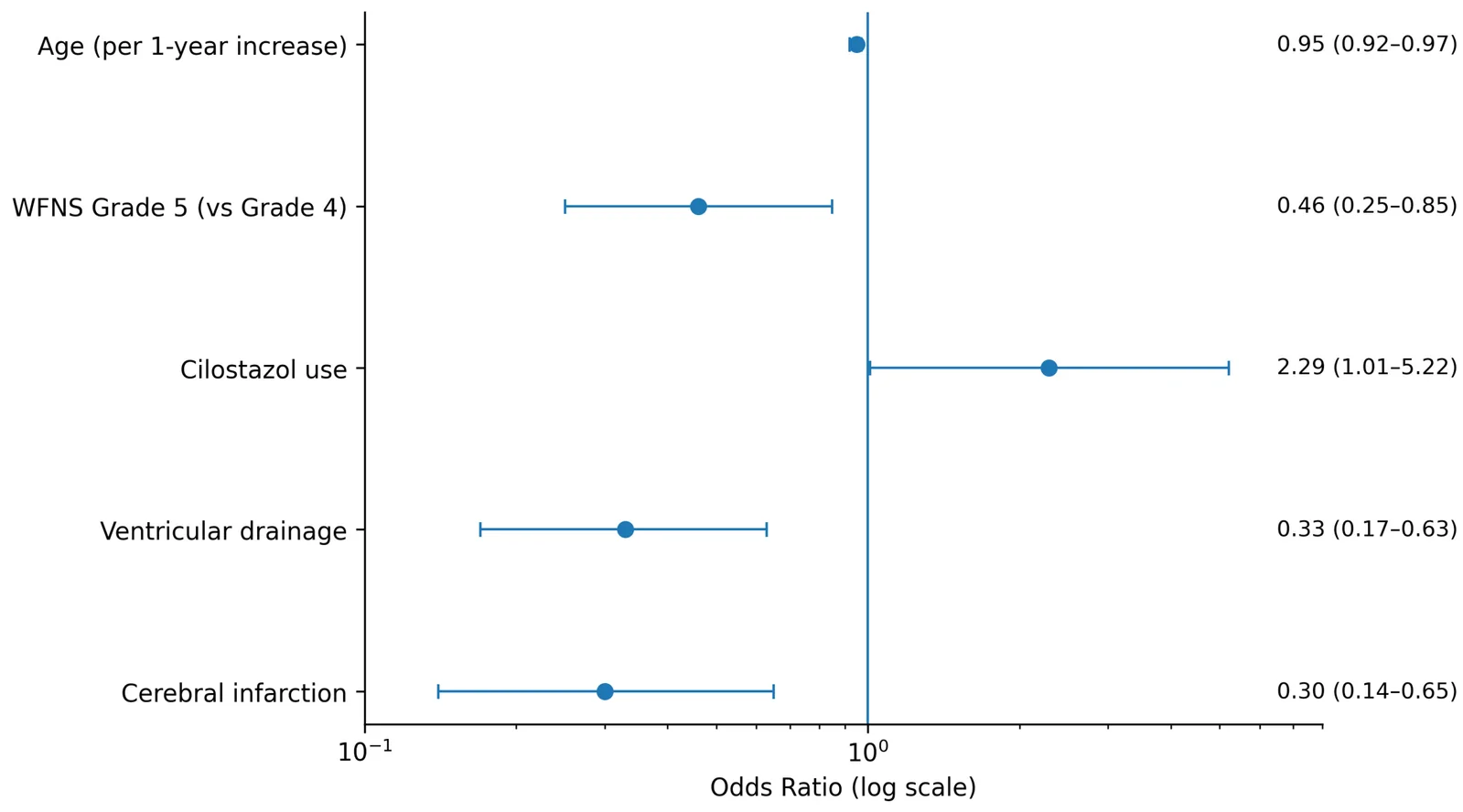
Forest plot of independent predictors of favorable functional outcome (mRS 0–2) at 6 months.

Univariate comparisons between patients with favorable and unfavorable outcomes are presented in Table 2. Six-month functional outcome data were available for 318 of the 394 enrolled patients (80.7%). Among these patients, 107 (33.6%) achieved a favorable outcome. When the entire enrolled cohort was used as the denominator, the proportion of patients with a confirmed favorable outcome was 27.2% (107/394). Compared with patients with available 6-month follow-up, those without available follow-up were older, more frequently had Fisher grade 4 and received cilostazol, and less frequently received clazosentan and intrathecal milrinone (Supplementary Table S3). These differences suggested that the missing outcome data could not be assumed to be completely random. Patients with favorable outcomes were significantly younger (60.8 ± 12.8 vs. 69.3 ± 13.2 years, *p* < 0.001) and less frequently classified as WFNS grade 5 (49.5% vs. 71.6%, *p* < 0.001). Treatment modality (clipping vs coiling) was not significantly associated with favorable outcome (*p* = 0.194). Cilostazol use was more common in the favorable outcome group than in the unfavorable outcome group (70.1% vs. 55.5%; *p* = 0.016). Ventricular drainage was less frequently performed in the favorable outcome group (29.0% vs. 47.9%; *p* = 0.002). Angiographic vasospasm occurred in 19 patients (17.8%) with favorable outcomes and 51 patients (24.2%) with unfavorable outcomes, with no significant between-group difference (*p* = 0.201). Symptomatic vasospasm occurred in 10 patients (9.4%) and 28 patients (13.3%), respectively, and was also not significantly different between the groups (*p* = 0.363). In contrast, cerebral infarction was significantly less frequent in patients with favorable outcomes than in those with unfavorable outcomes (15 patients [14.0%] vs. 61 patients [28.9%]; *p* = 0.003). Multivariable logistic regression analysis was conducted in 271 patients with complete data for all covariates (Table 3; Figure 2). Younger age (OR 0.95 per 1-year increase, 95% CI 0.92–0.97; *p* < 0.001) and cilostazol use (OR 2.29, 95% CI 1.01–5.22; *p* = 0.048) were independently associated with favorable outcome. Conversely, WFNS grade 5 (OR 0.46, 95% CI 0.25–0.85; *p* = 0.012), ventricular drainage (OR 0.33, 95% CI 0.17–0.63; *p* < 0.001), and cerebral infarction (OR 0.30, 95% CI 0.14–0.65; *p* = 0.002) were independently associated with lower odds of favorable recovery. No substantial multicollinearity was identified among the covariates included in the multivariable logistic regression model. VIF values ranged from 1.006 to 1.051, with all values well below the threshold of 5. The model demonstrated good discriminative performance, with an area under the receiver operating characteristic curve (AUC) of 0.802. In the mixed-effects logistic regression model accounting for center-level clustering, cilostazol use remained associated with favorable 6-month functional outcome (OR 2.35, 95% CI 1.02–5.38; *p* = 0.044; Supplementary Table S1). The direction and magnitude of the association were consistent with those observed in the primary conventional logistic regression model. In the multiple-imputation analysis, the estimated association between cilostazol use and favorable outcome remained directionally consistent with the primary analysis, although the confidence interval included the null (OR 1.58, 95% CI 0.89–2.82; *p* = 0.120). In the conservative boundary analysis in which all 76 patients with missing outcomes were classified as having an unfavorable outcome, the corresponding estimate was attenuated but remained in the same direction (OR 1.30, 95% CI 0.74–2.26; *p* = 0.360). The propensity score analyses included 271 patients, of whom 177 received cilostazol and 94 did not. Propensity score weighting substantially improved the balance of measured baseline covariates between the cilostazol and no-cilostazol groups, with all post-weighting absolute standardized mean differences below 0.10 (Supplementary Figure S1). In the ATT-weighted analysis, the estimated association between cilostazol use and favorable 6-month functional outcome remained directionally favorable, although the confidence interval included the null (OR 1.65, 95% CI 0.91–2.99; *p* = 0.101). The ATE-weighted analysis yielded a similar estimate (OR 1.61, 95% CI 0.91–2.86; *p* = 0.103).

Among the 271 patients included in the complete-case multivariable analysis, the distribution of DCI-preventive medication combinations and the corresponding favorable 6-month functional outcome rates are presented in Supplementary Table S2. Cilostazol was most frequently administered in combination with fasudil or clazosentan. Favorable outcome was observed in 39 of 93 patients (41.9%) receiving fasudil plus cilostazol, 27 of 61 patients (44.3%) receiving cilostazol plus clazosentan, and 5 of 21 patients (23.8%) receiving fasudil, cilostazol, and clazosentan. Cilostazol monotherapy was used in only two patients, precluding a meaningful subgroup comparison. Because several treatment combinations included small numbers of patients, the descriptive outcome rates were not subjected to formal between-group comparisons. In the additional multivariable analysis adjusting for concomitant fasudil, clazosentan, and intrathecal milrinone use, cilostazol remained associated with favorable 6-month functional outcome (OR 2.42, 95% CI 1.08–5.45; *p* = 0.032).

## 4. Discussion

This multicenter retrospective cohort study provides contemporary real-world data on the prognosis of poor-grade aneurysmal SAH. Despite the historically dismal outlook associated with WFNS grades 4–5, 33.6% of patients with available follow-up achieved functional independence at 6 months, underscoring that meaningful recovery remains attainable in a substantial proportion of patients under modern multimodal neurocritical care.

In the primary multivariable analysis, cilostazol use was associated with favorable functional outcome (OR 2.29, 95% CI 1.01–5.22; *p* = 0.048). However, the confidence interval was wide, and the association was attenuated and not statistically significant in propensity score–weighted and missing-outcome sensitivity analyses. These findings therefore suggest a potential signal but should not be interpreted as definitive evidence of treatment efficacy.

### 4.1. Indication Bias and Reverse Causation

We acknowledge the potential for indication bias, whereby patients with relatively preserved systemic condition might be more likely to receive enteral medication. In the participating centers, early enteral administration via nasogastric tube was routinely pursued after aneurysm treatment, even in comatose patients, provided no specific gastrointestinal contraindications were present. This practice likely reduces, but does not eliminate, the possibility that cilostazol exposure simply reflected preserved oral intake capacity.

Importantly, the association between cilostazol use and favorable outcome remained statistically significant in the primary multivariable analysis after adjustment for variables reflecting overall disease severity—including age, WFNS grade, ventricular drainage, and cerebral infarction. However, residual confounding and treatment-selection bias cannot be excluded. In addition, detailed patient-level data regarding treatment timing and duration were unavailable; therefore, exposure–response relationships and the optimal timing or duration of cilostazol therapy could not be evaluated. Because cilostazol was generally initiated after aneurysm securing, the observed association may have been affected by immortal time bias. A valid landmark or time-dependent analysis requires exact patient-level information regarding treatment initiation and changes in exposure status over time; however, these data were not systematically recorded in the registry. Consequently, immortal time bias could not be analytically eliminated, and the observed association should not be interpreted as evidence of a causal treatment effect. The estimated association between cilostazol use and favorable outcome remained in the same direction in analyses addressing missing 6-month outcomes, although the confidence intervals included the null. The loss of statistical significance may reflect both potential attrition bias and reduced precision resulting from uncertainty regarding the missing outcomes. Accordingly, the primary complete-case finding should not be interpreted as definitive evidence of treatment efficacy. Rather, the directionally consistent estimates across analyses should be regarded as hypothesis-generating and support further prospective evaluation of cilostazol in poor-grade aneurysmal subarachnoid hemorrhage. Propensity score weighting improved the balance of measured baseline covariates and yielded effect estimates in the same direction as the primary multivariable analysis, although the estimates were attenuated and the confidence intervals included the null. These findings suggest a potentially favorable association but do not provide definitive evidence of a treatment effect. The wider confidence intervals indicate uncertainty regarding the precision of the estimated association after adjustment for measured treatment-selection factors. The estimated association between cilostazol use and favorable functional outcome remained after additional adjustment for concomitant fasudil, clazosentan, and intrathecal milrinone use. However, cilostazol was generally administered as part of a multimodal DCI-prevention strategy, and cilostazol monotherapy was extremely uncommon. Therefore, the present findings do not isolate the pharmacological effect of cilostazol alone and should instead be interpreted as an association observed in the context of combined preventive therapy.

### 4.2. Impact of Cilostazol

Cilostazol, a phosphodiesterase-3 inhibitor, increases intracellular cyclic AMP, promoting vasodilation and inhibiting platelet aggregation [9]. Previous randomized studies and meta-analyses have demonstrated reductions in symptomatic vasospasm and DCI with cilostazol therapy [10,11]. However, data specific to poor-grade SAH remain limited, as such patients are frequently underrepresented in prospective trials [10,11,12].

Beyond macrovascular vasospasm, poor-grade SAH is characterized by profound microcirculatory dysfunction, endothelial injury, platelet activation, and inflammatory cascades contributing to early brain injury [4,8]. The potential benefit of cilostazol may therefore extend beyond vasospasm prevention. Through its pleiotropic effects—including vasodilation, antiplatelet activity, and endothelial modulation—cilostazol may target microvascular pathophysiology that is particularly pronounced in severe SAH [4,8,9]. This broader neurovascular profile may partly account for the association with functional recovery observed in the present cohort.

Future prospective studies with standardized treatment exposure and time-dependent assessment are warranted to further clarify causality.

### 4.3. Other Prognostic Factors

Consistent with prior literature, age and initial neurological severity remained strong predictors of outcome [2]. The distinction between WFNS grade 4 and 5 was clinically meaningful, with grade 5 patients demonstrating significantly reduced odds of favorable recovery. Ventricular drainage was independently associated with unfavorable outcome, likely reflecting the severity of intraventricular hemorrhage and acute hydrocephalus rather than a direct detrimental effect of the procedure itself.

Interestingly, although clazosentan and fasudil are widely used in Japan for vasospasm prevention [6], neither was significantly associated with favorable outcome in the univariable analysis. These findings should not be interpreted as evidence of therapeutic ineffectiveness of these agents, both of which have demonstrated vasospasm reduction in prior trials [7,18]. In particular, the present study was not designed to evaluate the efficacy of clazosentan. During the study period, clazosentan was introduced relatively recently, and its use may have varied according to institutional protocols, time period, patient severity, and perceived risk of adverse events such as fluid retention, pulmonary complications, or hypotension. Therefore, substantial confounding by indication and temporal bias may have influenced the observed association between clazosentan use and functional outcome. Further studies specifically designed to evaluate clazosentan in poor-grade SAH are warranted.

Overall, these findings highlight the complex and multifactorial determinants of long-term recovery in poor-grade SAH, in which modulation of angiographic vasospasm alone may be insufficient to improve functional outcomes [2,4,7].

### 4.4. Limitations

Several limitations warrant consideration. First, the retrospective design precludes causal inference and introduces the potential for selection bias and unmeasured confounding. Cilostazol exposure was defined as documented administration as part of DCI prophylaxis during the index hospitalization. Detailed patient-level data regarding the exact initiation date, treatment duration, cumulative dose, and discontinuation timing were unavailable. Consequently, treatment exposure intensity and exposure–response relationships could not be assessed, and some degree of exposure misclassification remains possible. The present findings therefore do not establish the optimal timing, duration, or cumulative dose of cilostazol therapy. Because exact patient-level dates of cilostazol initiation and discontinuation were unavailable, a valid landmark or time-dependent exposure analysis could not be performed, and immortal time bias cannot be excluded. Although non-randomized allocation limits causal interpretation, early enteral administration via nasogastric tube was routinely pursued across participating centers, including in comatose patients when no gastrointestinal contraindications were present. This protocol-driven practice may reduce the likelihood that cilostazol exposure merely reflected preserved swallowing function or systemic stability. Although propensity score weighting was performed to address confounding by indication, residual and unmeasured confounding cannot be excluded. Treatment selection may have been influenced by factors not systematically captured in the registry, including clinician judgment, contraindications, neurological trajectory after admission, exact treatment timing, concomitant therapies, and institutional preferences. In addition, several institutions had limited overlap in cilostazol use, preventing reliable inclusion of study centers directly in the propensity score model. Center-level clustering was evaluated separately using a mixed-effects logistic regression model. The propensity score–weighted estimates remained directionally favorable but their confidence intervals included the null; therefore, the observed association should be interpreted cautiously and regarded as hypothesis-generating. Cilostazol was frequently combined with other DCI-preventive therapies, and cilostazol monotherapy was rare. Although the additional multivariable analysis adjusted for concomitant fasudil, clazosentan, and intrathecal milrinone use, residual confounding related to specific treatment combinations, treatment timing, dose, duration, clinician selection, and institutional practice cannot be excluded. Consequently, the independent effect of cilostazol monotherapy could not be determined. In addition, the effect of clazosentan could not be adequately assessed because its use was influenced by institutional protocols, the timing of drug introduction during the study period, patient severity, and perceived risks of adverse events; therefore, the absence of an independent association with functional outcome should not be interpreted as evidence of lack of efficacy. Second, six-month functional outcome data were unavailable for 19.3% of the enrolled cohort, and patients without available follow-up differed from those with documented outcomes in several clinical and treatment characteristics. Although multiple-imputation and conservative boundary analyses were performed, residual attrition bias and uncertainty related to the missing outcomes cannot be eliminated. Statistical significance was not retained in these sensitivity analyses, although the effect estimates remained directionally consistent with the primary analysis. The observed association should therefore be interpreted cautiously. Third, although the multivariable model demonstrated good discriminative performance (AUC 0.802), external validation was not performed. Additionally, cerebral infarction was included as a covariate in the multivariable model; therefore, complex mediation pathways between cilostazol use, infarction, and outcome cannot be fully disentangled in this analysis. Although infarctions considered attributable to surgical or endovascular procedural complications were excluded based on the available imaging and clinical records, the remaining cerebral infarctions could not be reliably classified according to their underlying mechanism. Formal mediation analysis was not performed because the exact timing of cilostazol initiation, symptomatic vasospasm, and cerebral infarction was not systematically recorded, and the mechanisms of cerebral infarction could not be reliably classified. Therefore, the present study cannot determine whether the observed association was mediated through reductions in vasospasm or cerebral infarction. The generalizability of the present findings may be limited because DCI prevention and treatment strategies in Japan differ from those used in other healthcare systems. Fasudil and intrathecal milrinone are used in selected Japanese institutions but are not routinely used for this indication in several countries. Furthermore, the selection, combination, initiation, and duration of pharmacological therapies were not standardized across the participating institutions. Therefore, the present findings should be interpreted within the context of contemporary Japanese clinical practice and require validation in populations treated under different therapeutic protocols. In addition, the study population was restricted to patients with WFNS grades 4–5; therefore, the findings should not be generalized to patients with good-grade SAH. Despite these limitations, the multicenter design and relatively large sample size provide valuable real-world evidence regarding poor-grade aneurysmal SAH.

## 5. Conclusions

In this multicenter Japanese cohort of patients with poor-grade aneurysmal SAH classified as WFNS grades 4–5, cilostazol use was associated with favorable 6-month functional outcome in the primary multivariable analysis. However, this association was not statistically significant in several sensitivity analyses, including propensity score–weighted and missing-outcome analyses. These findings suggest a potential therapeutic signal in this specific high-risk population but should be interpreted as exploratory and should not be generalized to patients with good-grade SAH. Prospective studies with standardized treatment exposure and complete follow-up are warranted to further evaluate the potential role of cilostazol as an adjunctive DCI-prevention strategy in poor-grade aneurysmal SAH.

## Figures and Tables

**Table 1:** Baseline Characteristics of Patients with Poor-Grade SAH (*n* = 394).

Characteristics	Value
Age, mean ± SD ^1^ (years)	67.2 ± 13.6
Female sex, *n* (%)	272 (69.0%)
WFNS ^2^ Grade	
Grade 4, *n* (%)	136 (34.5%)
Grade 5, *n* (%)	258 (65.5%)
Fisher Grade 4, *n* (%)	147 (37.3%)
Comorbidities	
Hypertension, *n* (%)	163 (41.4%)
Diabetes Mellitus, *n* (%)	35 (8.9%)
Treatment Modality	
Surgical Clipping, *n* (%)	210 (53.3%)
Endovascular Coiling, *n* (%)	184 (46.7%)
DCI ^3^ Prevention	
Cilostazol Use, *n* (%)	250 (63.5%)
Fasudil Use, *n* (%)	231 (58.6%)
Clazosentan Use, *n* (%)	142 (36.0%)
Ventricular Drainage, *n* (%)	162 (41.1%)
Lumbar Drainage, *n* (%)	134 (34.0%)

Percentages for DCI prevention therapies are not mutually exclusive, as patients may have received more than one agent. ^1^ SD, Standard Deviation; ^2^ WFNS, World Federation of Neurosurgical Societies; ^3^ DCI, delayed cerebral ischemia.

**Table 2:** Univariate analysis of factors associated with favorable outcome (mRS 0–2).

Variable	Favorable Outcome (*n* = 107)	Unfavorable Outcome (*n* = 211)	*p*-Value
Age, mean ± SD ^1^ (years)	60.8 ± 12.8	69.3 ± 13.2	<0.001
WFNS ^2^ Grade 5, *n* (%)	53 (49.5%)	151 (71.6%)	<0.001
Fisher Grade 4, *n* (%)	28 (26.2%)	79 (37.4%)	0.059
Hypertension, *n* (%)	35 (32.7%)	93 (44.1%)	0.081
Treatment Modality			0.194
Surgical Clipping, *n* (%)	51 (47.7%)	117 (55.5%)	
Endovascular Coiling, *n* (%)	56 (52.3%)	94 (44.5%)	
DCI ^3^ Prevention			
Cilostazol Use, *n* (%)	75 (70.1%)	117 (55.5%)	0.016
Fasudil Use, *n* (%)	70 (65.4%)	116 (55.0%)	0.096
Clazosentan Use, *n* (%)	45 (42.1%)	80 (37.9%)	0.553
Ventricular Drainage, *n* (%)	31 (29.0%)	101 (47.9%)	0.002
Angiographic vasospasm, *n* (%)	19 (17.8%)	51 (24.2%)	0.201
Symptomatic vasospasm, *n* (%)	10 (9.4%)	28 (13.3%)	0.363
Cerebral Infarction, *n* (%)	15 (14.0%)	61 (28.9%)	0.003

^1^ SD, Standard Deviation; ^2^ WFNS, World Federation of Neurosurgical Societies; ^3^ DCI, delayed cerebral ischemia.

**Table 3:** Multivariable logistic regression analysis for favorable outcome (mRS 0–2).

Variable	Odds Ratio (95% CI ^1^)	*p*-Value
Age (per year increase)	0.95 (0.92–0.97)	<0.001
WFNS ^2^ Grade 5 (vs. Grade 4)	0.46 (0.25–0.85)	0.012
Cilostazol Use	2.29 (1.01–5.22)	0.048
Ventricular Drainage	0.33 (0.17–0.63)	<0.001
Cerebral Infarction	0.30 (0.14–0.65)	0.002

Model AUC: 0.802. ^1^ CI: Confidence Interval. ^2^ WFNS, World Federation of Neurosurgical Societies.

## Data Availability

De-identified data are available from Dr. Fusao Ikawa, who is responsible for data management, upon reasonable request, in accordance with the policy of the Shimane Prefectural Central Hospital Review Board.

## Supplementary Materials

The following supporting information can be downloaded at: https://www.mdpi.com/article/10.3390/jcm15166386/s1, Figure S1: Covariate balance before and after propensity score weighting; Table S1: Mixed-Effects Logistic Regression Analysis for Favorable 6-Month Outcome, with Study Centre as a Random Intercept; Table S2: DCI-preventive medication combinations and favorable 6-month functional outcomes; Table S3: Comparison of Patients With and Without Available 6-Month Functional Outcome Data.

## Abbreviations

The following abbreviations are used in this manuscript:

SAH	Subarachnoid Hemorrhage
WFNS	World Federation of Neurosurgical Societies
DCI	Delayed Cerebral Ischemia
EBI	Early Brain Injury
mRS	Modified Rankin Scale
CT	Computed Tomography
CSF	Cerebrospinal Fluid
OR	Odds Ratio
CI	Confidence Interval
AUC	Area Under the Curve
ROC	Receiver Operating Characteristic
VIF	Variance Inflation Factor
ATT	Average Treatment Effect among the Treated
ATE	Average Treatment Effect
