# Supplementary material for: Cilostazol and Functional Outcomes in Poor-Grade Aneurysmal Subarachnoid Hemorrhage: A Multicenter Retrospective Analysis
Source: J Clin Med. 2026 Aug 18;15(16):6386. doi: 10.3390/jcm15166386 (PMC13513675; doi:10.3390/jcm15166386)

Supplementary Figure S1. Covariate balance before and after propensity score weighting. Absolute standardized mean differences for the covariates included in the propensity score model are shown before weighting and after weighting targeting the average treatment effect among the treated (ATT) and the average treatment effect in the overall study population (ATE). The dashed vertical line indicates an absolute standardized mean difference of 0.10. After weighting, all covariates had absolute standardized mean differences below 0.10, indicating acceptable covariate balance.

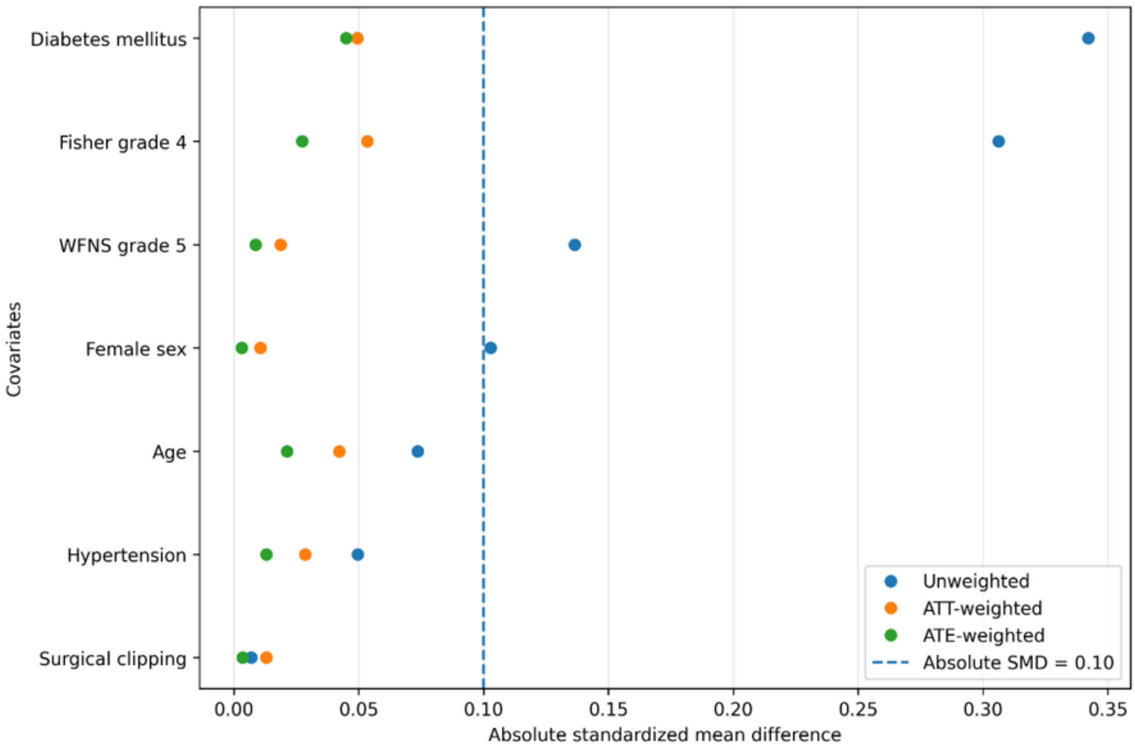

Supplement: Supplementary file 1 [file jcm-15-06386-s001.zip › Supplementary Figure S1.pdf]
