# Supplementary material for: Cilostazol and Functional Outcomes in Poor-Grade Aneurysmal Subarachnoid Hemorrhage: A Multicenter Retrospective Analysis
Source: J Clin Med. 2026 Aug 18;15(16):6386. doi: 10.3390/jcm15166386 (PMC13513675; doi:10.3390/jcm15166386)
Supplement: Supplementary file 1 [file jcm-15-06386-s001.zip › Supplementary Table S1.pdf]

Supplementary Table S1. Mixed-Effects Logistic Regression Analysis for Favorable 6-Month Outcome, with Study Centre as a Random Intercept

**Variable**	**Odds Ratio (95% CI)**	**p-value**
Age (per year increase)	0.95 (0.92–0.97)	< 0.001
WFNS Grade 5	0.34 (0.19–0.64)	< 0.001
Cilostazol Use	2.35 (1.02–5.38)	0.044
Ventricular Drainage	0.51 (0.26–0.99)	0.048
Cerebral Infarction	0.41 (0.19–0.88)	0.022

The model included study centre as a random intercept.

Abbreviations: CI, confidence interval; WFNS, World Federation of Neurosurgical Societies.
