# Supplementary material for: Cilostazol and Functional Outcomes in Poor-Grade Aneurysmal Subarachnoid Hemorrhage: A Multicenter Retrospective Analysis
Source: J Clin Med. 2026 Aug 18;15(16):6386. doi: 10.3390/jcm15166386 (PMC13513675; doi:10.3390/jcm15166386)
Supplement: Supplementary file 1 [file jcm-15-06386-s001.zip › Supplementary Table S2.pdf]

Supplementary Table S2. DCI-preventive medication combinations and favorable 6-month functional outcomes

**Medication combination**	**Total, *n***	**Favorable outcome, *n* (%)**
Fasudil + Cilostazol	93	39 (41.9%)
Cilostazol + Clazosentan	61	27 (44.3%)
Fasudil	26	6 (23.1%)
Fasudil + Intrathecal milrinone	23	12 (52.2%)
Fasudil + Cilostazol + Clazosentan	21	5 (23.8%)
Clazosentan	17	5 (29.4%)
None of these four agents	16	0 (0%)
Clazosentan + Intrathecal milrinone	4	1 (25.0%)
Fasudil + Clazosentan	4	1 (25.0%)
Intrathecal milrinone	3	0 (0%)
Cilostazol	2	0 (0%)
Fasudil + Clazosentan + Intrathecal milrinone	1	0 (0%)
**Total**	**271**	**96 (35.4%)**

Values are presented as n or n (%).
The analysis included 271 patients with available 6-month functional outcome data and complete information for the covariates used in the multivariable analysis. Favorable functional outcome was defined as a modified Rankin Scale score of 0–2 at 6 months. Medication combinations were classified according to documented use of fasudil, cilostazol, clazosentan, and intrathecal milrinone during the index hospitalization. Because several treatment combinations included very small numbers of patients, these descriptive rates were not subjected to formal between-group comparisons.

Abbreviations: DCI, delayed cerebral ischemia; mRS, modified Rankin Scale.
