# Supplementary material for: Cilostazol and Functional Outcomes in Poor-Grade Aneurysmal Subarachnoid Hemorrhage: A Multicenter Retrospective Analysis
Source: J Clin Med. 2026 Aug 18;15(16):6386. doi: 10.3390/jcm15166386 (PMC13513675; doi:10.3390/jcm15166386)
Supplement: Supplementary file 1 [file jcm-15-06386-s001.zip › Supplementary Table S3.pdf]

Supplementary Table S3. Comparison of Patients With and Without Available 6-Month Functional Outcome Data.

**Variable**	**Outcome available (*n* = 318)**	**Outcome unavailable (*n* = 76)**	**p-value**
Age, mean ± SD (years)	66.5 ± 13.7	70.5 ± 12.6	0.014
Female sex, *n* (%)	222 (69.8%)	50 (65.8%)	0.496
WFNS grade 5, *n* (%)	204 (64.2%)	54 (71.1%)	0.256
Fisher grade 4, *n* (%)	107 (33.6%)	40 (52.6%)	0.002
Hypertension, *n* (%)	128 (43.0%)	35 (46.7%)	0.562
Diabetes mellitus, *n* (%)	30 (10.0%)	5 (6.6%)	0.355
Surgical clipping, *n* (%)	168 (52.8%)	42 (55.3%)	0.703
Cilostazol use, *n* (%)	192 (60.4%)	58 (76.3%)	0.010
Fasudil use, *n* (%)	186 (58.5%)	45 (59.2%)	0.909
Clazosentan use, *n* (%)	125 (39.3%)	17 (22.4%)	0.006
Intrathecal milrinone use, *n* (%)	39 (12.3%)	0 (0.0%)	0.001
Ventricular drainage, *n* (%)	132 (41.5%)	30 (39.5%)	0.746

Values are presented as mean ± standard deviation or n (%). Percentages for hypertension and diabetes mellitus were calculated using available data because these variables contained missing values. Between-group comparisons were performed using Welch's t-test for age and the chi-square test or Fisher's exact test, as appropriate, for categorical variables.

Abbreviations: DCI, delayed cerebral ischemia; SD, standard deviation; WFNS, World Federation of Neurosurgical Societies.
